# A Diagnostic Quandary of Escherichia Coli Pneumonia: A Case Report and Literature Review

**DOI:** 10.7759/cureus.39668

**Published:** 2023-05-29

**Authors:** Venu M Ganipisetti, Natasha Dudiki, Anand Athavale

**Affiliations:** 1 Hospital Medicine, Presbyterian Hospital, Albuquerque, USA; 2 Pulmonary and Critical Care, Indiana University Health Ball Memorial Hospital, Muncie, USA

**Keywords:** diabetes mellitus type 2, community-acquired pneumonia (cap), gram negative pneumonia, diverticulitis colon, expec, e coli: escherichia coli, e. coli pneumonia

## Abstract

*Escherichia coli* community-acquired pneumonia (CAP) is an under-recognized condition associated with higher mortality compared to the other well-studied causes of pneumonia. *E. coli* pneumonia is frequently associated with bacteremia. Despite the absence of abdominal or urinary symptoms, the infection may originate from an occult gastrointestinal (GI) source since it is a common commensal bacteria of the GI tract. Conditions related to extraintestinal pathogenic *E. coli* (ExPEC) are gaining attention, and there has been a trend toward the rise of pneumonia secondary to gram-negative bacteria. This presents a diagnostic stewardship dilemma in a patient with sepsis, *E. coli* bacteremia, and apparent pneumonia - to assume and treat for *E. coli* CAP or to look for a GI/genitourinary source which may, in turn, lead to incidental findings and further studies. We report a case of *E. coli *CAP in a 62-year-old patient and our approach regarding the treatment and imaging course.

## Introduction

*Escherichia coli* (*E. coli*), a Gram-negative bacteria, is a member of the family *Enterobacteriaceae *and is usually a commensal in the gastrointestinal (GI) tract of humans and animals. However, it can acquire mobile genetic elements that confer virulence factors and allow it to become a pathogen that can cause pneumonia, diarrhoea, enteritis, biliary tract infections, urinary tract infections, bacteremia, and neonatal meningitis [1]. *E. coli* pneumonia is usually thought of as a nosocomial infection occurring in patients with risk factors such as aspiration or mechanical ventilation [2,3]. In contrast, the propensity to cause community-acquired pneumonia (CAP) is under-recognized. In recent years, *E. coli* with extraintestinal pathogenicity, termed ExPEC, have gained attention and are postulated to cause a spectrum of infections, including bacteremia, urinary tract infections, prostate infections, pneumonia, liver abscess, meningitis, and brain abscesses [2]. We report a patient who presented with clinical and radiologic evidence of pneumonia and subsequent blood cultures revealing *E. coli* bacteremia without any abdominal or urinary symptoms. Due to the perpetual association of GI sources with *E. coli *infections, further venture investigations showed evidence of asymptomatic acute diverticulitis.

## Case presentation

A 62-year-old Caucasian female with a past medical history significant for asthma, type 2 diabetes mellitus, obesity, essential hypertension, hypothyroidism, and depression presented to the emergency room (ER) from home with symptoms of chills, congestion, rhinorrhea, nonproductive cough, anorexia, nausea, and subjective fevers for five days prior to presentation. She has no reported travel history, recent hospitalizations, or exposure to sick contacts. She does not smoke cigarettes, drink alcohol, or do recreational drugs. Home medications included insulin, empagliflozin, metformin, pioglitazone, fosinopril, levothyroxine, rosuvastatin, trazodone, escitalopram, and vitamin D. Allergies include codeine. Vitals in the ER revealed a temperature of 104.8 F, heart rate of 133 beats per minute, respiratory rate of 44 cycles per minute, blood pressure of 81/56 mm Hg, oxygen saturations in the low 80s, and she required six litres of supplemental oxygen to maintain her saturations adequately.

Initial labs are summarized in Table 1.

**Table 1:** Initial laboratory test results WBC: white blood cell count; Hb: haemoglobin; HbA1C: haemoglobin A1C; CO_2_: carbon dioxide; BUN: blood urea nitrogen

Lab (reference range)	Result
WBC (4.0-11.0 x10E3/uL)	12
Absolute neutrophil count (1.8-7.0 x10E3/uL)	10.9
Hb (12-16 gm/dL)	10.6
Platelets (150-400 x10E3/uL)	175
HbA1C (4.4-5.6%)	7.9%
Sodium (135-145 mmol/L)	130
Potassium (3.5-5.1 mmol/L)	4.5
Chloride ( 98-107 mmol/L)	96
CO_2_ (19-29 mmol/L)	14
Anion Gap (<18 mmol/L)	20
Glucose (60-100 mg/dL)	580
Creatinine (0.50-1.04 mg/dL)	2.71 (baseline renal function is normal)
BUN (7-25 mg/dL)	45
Albumin (3.3-5.2 gm/dL)	2.5
Lactic acid (0.5-2.2 mmol/L)	4.4
Procalcitonin (<0.10 ng/mL)	55.8
Betahydroxybutarate (<0.6 mmol/L)	2.5

**Figure 1:**
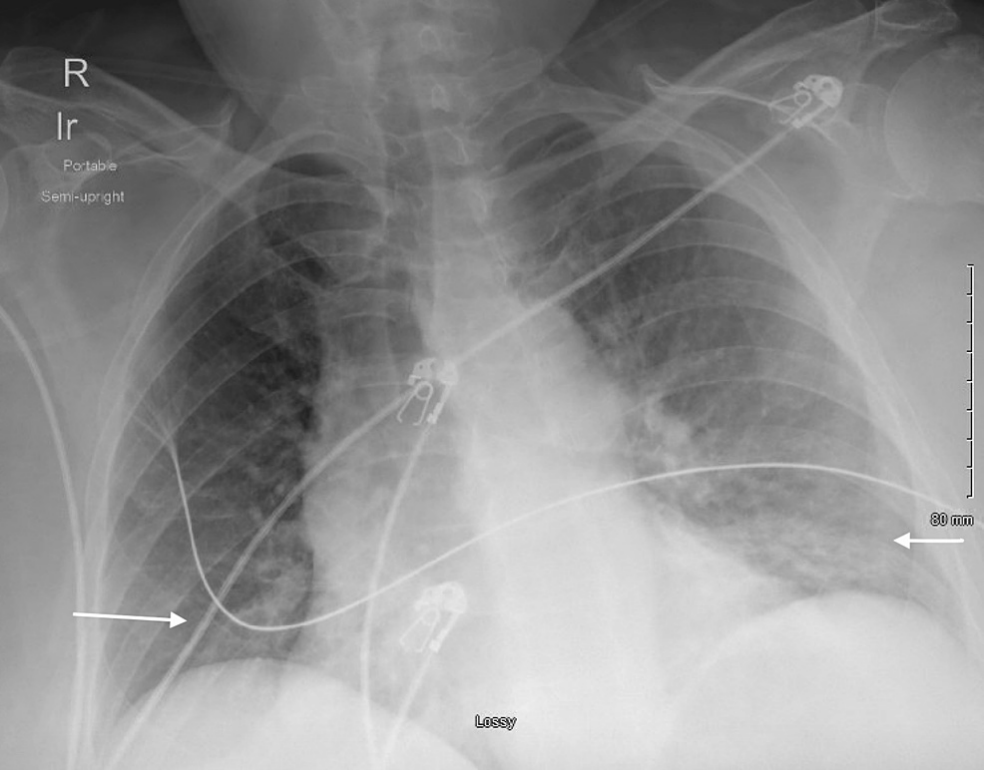
Chest X-ray showing bibasilar infiltrates.

**Figure 2:**
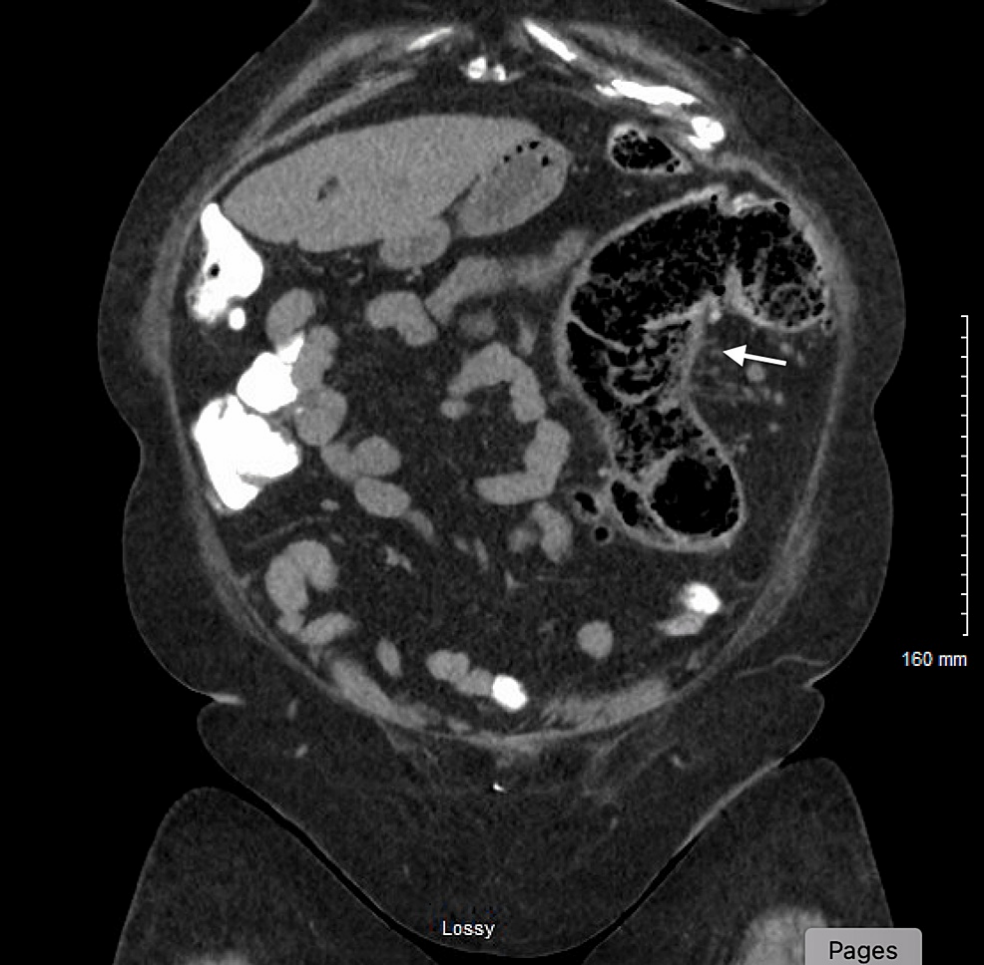
CT abdomen showing abnormal inflammatory changes suggestive of diverticulitis in the colon. CT: computed tomography

**Figure 3:**
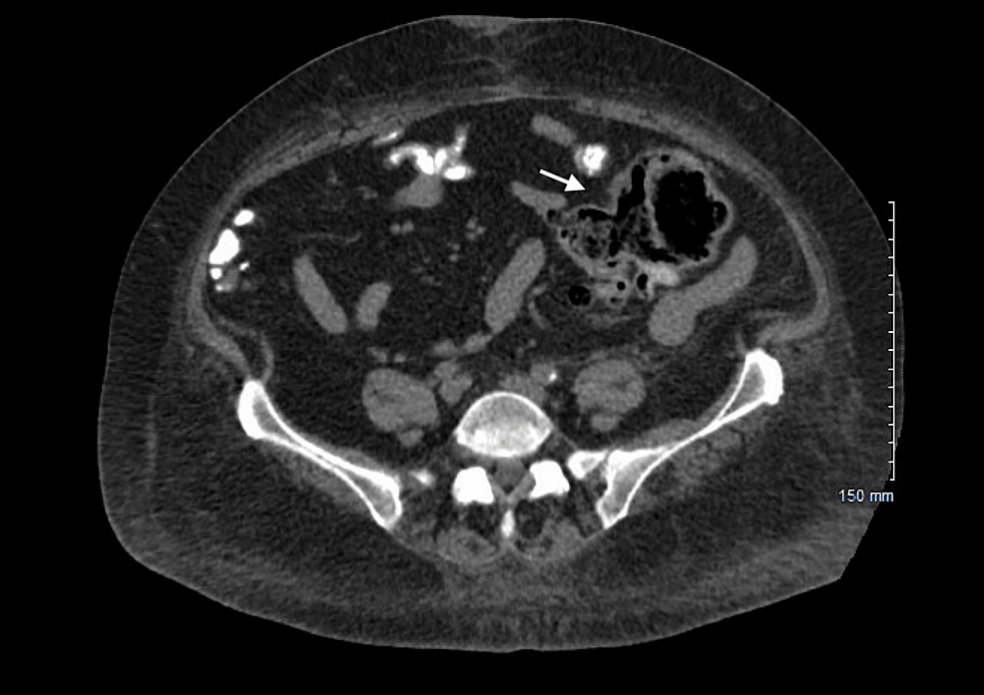
CT abdomen showing inflammatory changes in the colon. CT: computed tomography

Given her presenting symptoms, hypoxia, laboratory abnormalities showing leukocytosis, lactic acidosis, and elevated procalcitonin in the setting of chest X-ray showing bibasilar infiltrates (Figure 1), she was diagnosed with severe sepsis due to pneumonia. She was started on ceftriaxone (2 grams intravenous daily) and doxycycline (100 milligrams intravenous twice daily). She is also noted to have diabetic ketoacidosis (initial pH on blood gas was 7.31), which promptly improved with insulin therapy (insulin drip followed by a transition to subcutaneous insulin) and intravenous fluids. She has not required pressor support. Blood cultures collected upon arrival to the ER returned positive for *E. coli* (pan-sensitive except resistant to ampicillin, ampicillin/sulbactam, and amoxicillin/clavulanic acid). Urine cultures remained negative. Her initial hospital course was also complicated by an episode of transient wide complex sinus tachycardia with left bundle branch block morphology. 2D echo showed normal left ventricle (LV) function without resting regional wall motion abnormalities or hemodynamically significant valvular issues. A nuclear stress test was normal. The patient did not endorse any urinary or abdominal symptoms throughout her hospital course, including abdominal pain, vomiting, and diarrhoea. Due to high suspicion of an occult GI source responsible for *E. coli* bacteremia and pneumonia, computed tomography (CT) abdomen pelvis was undertaken, which showed findings of colonic diverticulosis with peri diverticular inflammatory changes surrounding the descending and rectosigmoid colon (Figures 2, 3). No associated abscess or bowel perforation was noted. She reported having had a normal colonoscopy five years ago. She continued to improve clinically and was discharged on oral sulfamethoxazole-trimethoprim (800-160 mg tablets, one tablet twice daily) to complete a 14-day course total duration of antibiotics. Her room air saturations were around 87%, and she required one litre of supplemental oxygen upon discharge. We arranged an outpatient referral to follow up with gastroenterology for an elective colonoscopy to exclude the possibility of underlying intestinal malignancy.

## Discussion

*E. coli *is a non-spore-forming Gram-negative bacillus belonging to the *Enterobacteriaceae *family, now known as *Enterobacterales*. It is a common commensal organism found in the human gut. It is also found in the gut of cattle, sheep, and goats [4].

In the United States, *E. coli* is responsible for 265,000 illnesses and about 100 deaths annually [5]. Clinical syndromes of pathogenic *E. coli* usually include diarrhoea/dysentery, biliary tract infections, urinary tract infections, and meningitis [6]. *E. coli* variants responsible for extraintestinal infections have been labelled ExPEC. The evolution, pathophysiology, and drug resistance mechanisms of pathogenic *E. coli* are poorly understood. The true incidence of *E. coli *pneumonia is still debated and unknown. The incidence of *E. coli* pneumonia is reported to be around 3-12% of CAP infections [6]. Identifying true incidence could be challenging for multiple reasons, including that patients treated for CAP only sometimes have collected blood or sputum cultures. In a multicenter study of 2259 patients (EPIC study - aetiology of pneumonia in the community), performed by Jain et al. to identify the incidence of pathogens causing CAP in patients with available microbiological specimens for testing and had radiological evidence of pneumonia, a pathogen was identified in only 38% of cases. The most common pathogens were Rhinovirus (9%), influenza virus (6%), and *Streptococcus pneumonia* (5%). *Enterobacteriaceae *prevalence was less than 1%, with incidence more common in ICU patients than non-ICU patients. This study, however, has limitations in that severely immunosuppressed patients were excluded in whom *Enterobacteriaceae *might be more prevalent [7].

A large retrospective cohort study from 173 hospitals reported *E. coli* pneumonia constituted 7.7% of culture-positive Gram-negative pneumonia. It was interestingly noted in this study that despite confirmation of *E. coli *pneumonia, antibiotics were not narrowed down in many instances, indicating that the physician community still does not widely consider *E. coli* as a causative agent of pneumonia. However, some of the limitations of this study include that this is a retrospective study, and out of 171709 patients with a presumed diagnosis of pneumonia, only 8680 patients who had culture confirmation were included for analysis. The included patient population was also based on positive respiratory cultures. Hence, actual infection vs. colonization was challenging to exclude [6]. There has been a recent increase in the incidence of Gram-negative bacteria pneumonia. Most cases of *E. coli* pneumonia are related to ventilator-associated pneumonia and have surpassed *Pseudomonas aeruginosa *and *Staphylococcus aureus* in recent years. A study published in 2019 by La Combe et al. described 260 *E. coli* isolates from 243 ICU patients, with 117 patients diagnosed with ventilator-associated pneumonia, 61 with hospital-acquired pneumonia, and 82 with CAP [8]. Patients with *E. coli* pneumonia have been reported to be older (median age of 76 years) and have more comorbidities than patients with pneumococcal pneumonia [6]. *E. coli* pneumonia is also noted to have male predominance [9].

Implicated mechanisms for *E. coli* pneumonia include aspiration and hematogenous dissemination due to bacteremia. Gut mucosal membrane disruption can lead to local tissue invasion from infection and subsequently lead to bacteremia, causing distant seeding and leading to infection outside of the GI tract. Aspiration or microaspiration of secretions is an alternate aetiology of pneumonia but usually happens in the settings of altered mental status, gastroesophageal reflux disease (GERD), or functional disorders in swallowing. Hence, this poses a diagnostic dilemma in patients similar to our patient in this case, without GI symptoms, negative urine cultures, and no obvious risk factors for aspiration. A review of case reports describes alcoholism, diabetes mellitus, and chronic lung or heart diseases as potential risk factors [9].

*E. coli* pneumonia is associated with significant mortality and morbidity. Previous studies have also reported that patients with *E. coli* pneumonia are frequently bacteremic [6]. A study published in 2021 by John et al. described significantly higher case fatality than patients with pneumococcal pneumonia (adjusted odds ratio, 1.55; 95% CI, 1.23-1.97). They also reported a higher incidence of bacteremia; nearly 40% of the patients needed ICU admission, 20% required respiratory support, and a 14% in-hospital case fatality rate [6]. The Pneumonia PORT study published in 1998 described 19 inpatients with *E. coli* pneumonia with associated bacteremia reported in 48%, 84% with a pneumonia severity index category of 4-5, which conferred expected mortality as high as 27% at 30 days. While the in-hospital case fatality was 0, the 90-day case fatality rate was 21% [10]. While therapy should be guided by the patient’s clinical status, history, risk factors, and local antibiogram, one should be mindful of the emergence of multi-drug resistance (MDR) in *E. coli*. According to data from the Centers for Disease Control (CDC), the prevalence of MDR *E. coli* strains has increased steadily over the past decade. In some states, such as California, Nevada, and Texas, the percentage of MDR *E. coli* strains exceeds 15%. A recent retrospective study of *E. coli* pneumonia reported that 54% of the isolates were resistant to ampicillin, 36% to respiratory fluoroquinolones, and 9% to ceftriaxone [6]. Cultures in our patient were resistant to ampicillin. We describe a case report of the patient presenting with clinical symptoms of pneumonia with subsequent laboratory data and chest X-ray confirming it. Her pneumonia severity index was calculated to be 4. She had associated *E. coli *bacteremia. Fortunately, she responded with expeditious initiation of antibiotics and was eventually discharged home. Her possible risk factors include diabetes mellitus and asthma. Since *E. coli *pneumonia is frequently associated with bacteremia with potential origin from a GI source, we pursued additional investigations with a CT of the abdomen pelvis despite the lack of any GI symptoms, which incidentally showed findings of colonic diverticulosis with peri diverticular inflammatory changes surrounding the descending and rectosigmoid colon. She was referred to an outpatient GI specialist for an elective colonoscopy, given her CT findings and to rule out underlying malignancy. Certain phylogenic groups of *E. coli* (B1, D) are known to release cyclomodulin toxins, which may interfere with cell cycle regulation, alter enterocytes, and increase susceptibility to malignancy [11]. In 2017, Patel et al. described a patient who underwent colonoscopy for *E. coli* bacteremia and pneumonia, which showed an ulcerated cecal mass measuring 2.6 × 1.8 × 0.5 cm with pathology showing moderately differentiated adenocarcinoma [11]. This highlights the challenges faced when encountering *E. coli *pneumonia with bacteremia. Further research may be warranted to evaluate the risks and benefits of additional screening evaluation to identify GI sources.

## Conclusions

Community-acquired *E. coli* pneumonia, a type of ExPEC, is an under-recognized entity and can pose a diagnostic dilemma in the absence of risk factors such as aspiration, recent hospitalization, or mechanical ventilation. Since *E. coli* is a common intestinal commensal organism and pneumonia is frequently associated with bacteremia, it is possible for an abdominal source, even without associated abdominal symptoms. By reporting this case, we emphasize the importance of awareness of the increasing incidence of gram-negative pneumonia such as *E. coli* pneumonia and the role of exploring possible abdominal sources in such cases using dedicated imaging. Asymptomatic diverticulitis or abdominal malignancy should be considered in the differential diagnosis, and early workup may be beneficial. However, on the same note, risks of unnecessary imaging, radiation exposure, and costs should be outweighed, and further research about the role of source exploration is warranted.
